# Genome-Wide Identification and Expression Analysis of the Aux/IAA and Auxin Response Factor Gene Family in *Medicago truncatula*

**DOI:** 10.3390/ijms221910494

**Published:** 2021-09-28

**Authors:** Rui Liu, Zhenfei Guo, Shaoyun Lu

**Affiliations:** 1 Guangdong Engineering Research Center for Grassland Science, State Key Laboratory for Conservation and Utilization of Subtropical Agro-Bioresources, College of Life Sciences, South China Agricultural University, Guangzhou 510642, China; 20181002003@stu.scau.edu.cn; 2 College of Grassland Science, Nanjing Agricultural University, Nanjing 210095, China

**Keywords:** abiotic stress, Aux/IAA, auxin response factor (ARF), *cis*-acting elements, *Medicago truncatula*

## Abstract

*Aux/IAA* and auxin response transcription factor (*ARF*) genes are key regulators of auxin responses in plants. A total of 25 *MtIAA* and 40 *MtARF* genes were identified based on the latest updated *Medicago truncatula* reference genome sequence. They were clustered into 10 and 8 major groups, respectively. The homologs among *M. truncatula*, soybean, and *Arabidopsis thaliana* shared close relationships based on phylogenetic analysis. Gene structure analysis revealed that *MtIAA* and *MtARF* genes contained one to four concern motifs and they are localized to eight chromosomes, except chromosome 6 without *MtARFs.* In addition, some *MtIAA* and *MtARF* genes were expressed in all tissues, while others were specifically expressed in specific tissues. Analysis of *cis*-acting elements in promoter region and expression profiles revealed the potential response of *MtIAA* and *MtARF* genes to hormones and abiotic stresses. The prediction protein–protein interaction network showed that some ARF proteins could interact with multiple Aux/IAA proteins, and the reverse is also true. The investigation provides valuable, basic information for further studies on the biological functions of *MtIAA* and *MtARF* genes in the regulation of auxin-related pathways in *M. truncatula*.

## 1. Introduction

Indole-3-acetic (IAA) is the primary auxin in higher plants and regulates plant growth and development as well as responses to environmental stimuli [1,2]. The changes in auxin levels trigger downstream gene reprogramming through auxin response genes, such as the *auxin/indole-3-acetic acid* (*Aux/IAA*) family, the *auxin response factor* (*ARF*) family, and *small auxin upregulated RNA* (*SAUR*), and the auxin-responsive *gretchen hagen 3* (*GH3*) family [3]. Aux/IAAs and ARFs are essential for auxin-mediated transcriptional regulation [4,5]. Aux/IAA proteins bind with ARFs for repressing activation of downstream auxin-responsive genes in the absence of auxin. Aux/IAA is ubiquitinated by interacting with TRANSPORT INHIBITOR RESPONSE 1/AUXIN SIGNALING F-BOX (TIR1/AFB) receptors and subsequently degraded via the 26S proteasome under high concentrations of auxin [4,5]; ARFs are released for regulation of the expression of auxin-responsive genes [4,5].

Twenty-nine Aux/IAA gene family members are found in *Arabidopsis* [6]. Four highly conserved domains exist in most Aux/IAA proteins. Domain I has a conserved leucine repeat (LXLXLX) motif that interacts with TOPLESS (TPL) protein (a co-repressor protein) to mediate auxin-dependent transcriptional repression [7]. Domain II is the auxin degron with a conserved “GWPPV” motif that directly interacts with SCF^TIR1^ (SKP1/Cullin/F-box protein complex containing the transport inhibitor response 1 protein) and is associated with the turnover of Aux/IAA proteins [8]. A carboxy-terminal PB1 (Phox and Bem 1) domain is contained within a region that was previously called domain III/IV and functions to interact with ARF. It is, thus, involved in the regulation of ARF activity [9]. Aux/IAA is involved in the regulation of diverse cellular and developmental processes, including embryogenesis, axis formation and patterning, lateral root initiation, leaf expansion, vascular elongation, tropism, inflorescence and fruit development, apical dominance, and defense responses against pathogens [10,11,12]. For example, loss in the function of *iaa3/shy2* mutation affects auxin homeostasis and the formation of lateral roots [13]. TIR1/AFB2 form specific sensing complexes with AtIAA6, AtIAA9 and/or AtIAA17 to modulate JA homeostasis and adventitious root initiation in the presence of auxin [14]. AtIAA33 maintains root distal stem cell identity and negatively regulates auxin signaling by interacting with AtARF10 and AtARF16 [15]. Aux/IAA family members are also identified in other plants, such as tomato (*Solanum lycopersicon*), cucumber (*Cucumis sativus*), maize (*Zea mays*), and rice (*Oryza sativa*) [16,17,18,19].

Twenty-three ARF members are identified in *Arabidopsis* [20]. Most ARF proteins are consisted of an N-terminal DNA-binding domain (DBD), a middle region (MR) and a C-terminal dimerization domain (CTD) [3]. The DBD belongs to B3-like family and enables ARFs to specifically bind with the TGTCTC auxin response elements (AuxREs) present in the promoters of various auxin-responsive genes [21]. The MR, whose sequence is less conserved, depends on ARF as a transcriptional activator or repressor depending on its amino acid composition [21,22]. The CTD domain is involved in homo- and heterointeraction and inhibits its binding to the auxin-responsive elements under low auxin concentration conditions [23]. Some *ARF* genes conferring diverse biological processes have been functionally characterized [10]. For instance, AtARF7 and AtARF19 proteins are essential for auxin-medicated plant development by regulating both unique and partially overlapping sets of target genes [20]. AtARF2-4 and AtARF5 are essential for female and male gametophyte development [24]. AtARF8 regulates stamen elongation and endothecium lignification [25], while AtARF3 plays a distinct role during early flower development [26]. Some ARF family members are also identified in other plants, such as soybean (*Glycine max*), maize, potato, and *Brachypodium distachyon* [27,28,29,30].

*Medicago truncatula* is a model legume due to its small genome, self-pollination and many seeds, high genetic transformation efficiency, and large number of mutants [31]. Genome-wide analysis of *Aux/IAA* and *ARF* revealed that there are 17 *Aux/IAA* and 24 *ARF* genes in *M.truncatula* based on version Mt3.5 of the *M. truncatula* genome database [32,33]. Most of the *MtIAA* and *MtARF* genes were expressed in response to the early phase of *S. meliloti* infection, revealing the distinctive expression and function features of *Aux/IAA* and *ARF* family genes in *Medicago trunctula* during nodule formation and symbiotic interaction, respectively [32,33]. However, 25 *Aux/IAA* and 40 *ARF* members were isolated when we searched in version Mt4.0v1 of the *M. truncatula* genome database; the effect of abiotic stress on MtIAA or MtARF and the prediction of the interaction between MtIAA and MtARF have not been reported. Given the important role of *Aux/IAA* and *ARF* in regulation on plant growth and development, it is worth updating the information. The objectives of this study were to analyze the gene structures, chromosomal locations, phylogenetic relationships, motif organization, *cis*-acting element and expression patterns under salt, drought and cold treatments, and predicted protein interaction network of *Aux/IAA* and *ARF* genes in *M. truncatula*. The results provide a comprehensive understanding of the *Auxin*/*IAA* and *ARF* gene families in *M. truncatula*.

## 2. Results

### 2.1. Identification of IAA and ARF Members in M. truncatula

The *IAA* and *ARF* genes of *Arabidopsis thaliana* and soybean were used as query sequences to search for *IAA* and *ARF* genes in the genomes of *M. truncatula*. Twenty-five *MtIAA* and 40 *MtARF* genes were obtained, and their deduced peptides were confirmed after domain analysis, using Pfam and SMART databases. A total of 25 and 40 members were finally identified, and they were named *MtIAA1* to *MtIAA25* (Table 1) and *MtARF1* to *MtARF40* (Table 2), respectively, based on their locations on the chromosomes. The amino acid sequence of MtIAAs and MtARFs were further analyzed. The amino acids in length were ranged from 161 amino acids in MtIAA22 to 356 amino acids in MtIAA24, with an average of 237 in MtIAAs, while the average amino acid length of MtARFs was 642, ranging from 163 amino acids in MtARF29 to 1265 amino acids in MtARF1. The predicted molecular weight (MW) varied from 18.19 KDa to 38.67 KDa, and the theoretical isoelectric point (pI) varied from 4.77 to 9.00 in MtIAAs (Table 1), while the predicted MW varied from 18.06 KDa to 141.1 KDa, and pI varied from 5.14 to 8.87 in MtARFs (Table 2). The predicted grand average of hydropathicity (GRAVY) of all MtIAAs and MtARFs were negative, indicating that MtIAAs and MtARFs are hydrophilic proteins (Table 1 and Table 2).

### 2.2. Phylogenetic Analysis of IAAs and ARFs among M. truncatula, Soybean and Arabidopsis

**Figure 1:**
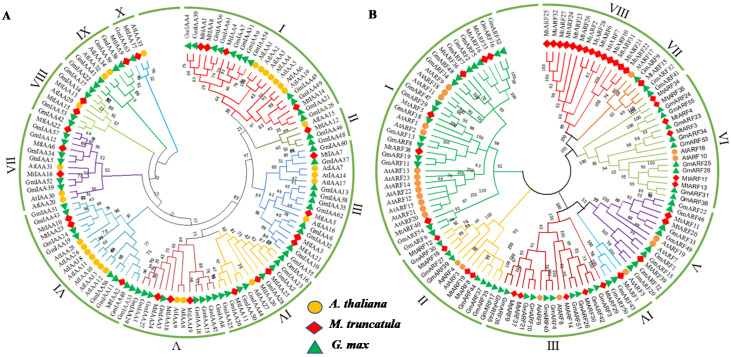
Phylogenetic trees of IAA (**A**) and ARF (**B**) proteins among *M. truncatula*, *Arabidopsis thaliana* and soybean. The tree was constructed with 1000 bootstrap replications. IAAs and ARFs from *A. thaliana*, *M. truncatula* and soybean were indicated using orange, red and green colors, respectively, for distinguishing them.

All IAA and ARF proteins in *M. truncatula* (25, 40), *Arabidopsis* (29, 23) and soybean (63, 55) were aligned to generate unrooted phylogenetic trees for evaluation of their evolutionary relationship (Figure 1A,B). The sequences of MtIAA and MtARF proteins are listed in Table S1. Referring to those in *Arabidopsis thaliana*, soybean, and *Brassica napus* [20,34,35], the Aux/IAA family was classified into two groups, A and B. Group A was subdivided into four subgroups (I to IV), containing 57 members (12 in *M. truncatula*, 13 in *Arabidopsis*, and 32 in soybean) (Figure 1A), while the group B was subdivided into six subgroups (V to X), containing 60 members (13 in *M. truncatula*, 16 in *Arabidopsis*, and 31 in soybean) (Figure 1B). Each subgroup contained the Aux/IAA members of the above three species, indicating that the Aux/IAA differentiation time was earlier than the species differentiation. In addition, the phylogenetic tree showed that Aux/IAA members in *M. truncatula* were closely related to those in soybean.

ARFs were clustered into eight groups (I to VIII). According to the amino acid sequence of MR in the middle region of MtARF, MtARF members could be divided into transcriptional activators and repressors. MtARF1, -9, -11, -14, -20, -29, -30, -37 and -39 were predicted to be transcription activators containing a Q-, S-, and L-rich MR domain in subclasses III to V, while MtARF5, -16, -33, -36, -38 and -40 containing a S-, P-, L-, and G-rich MR domain were predicted to be transcription repressors in subclass I. The MtARF members from subclasses I, II and IV–VII were more closely related to those in soybean and *Arabidopsis* than those in subclass VIII, indicating a trend in the development of ARF family members across different plant species.

### 2.3. Gene Structure and Domain Architecture

**Figure 2:**
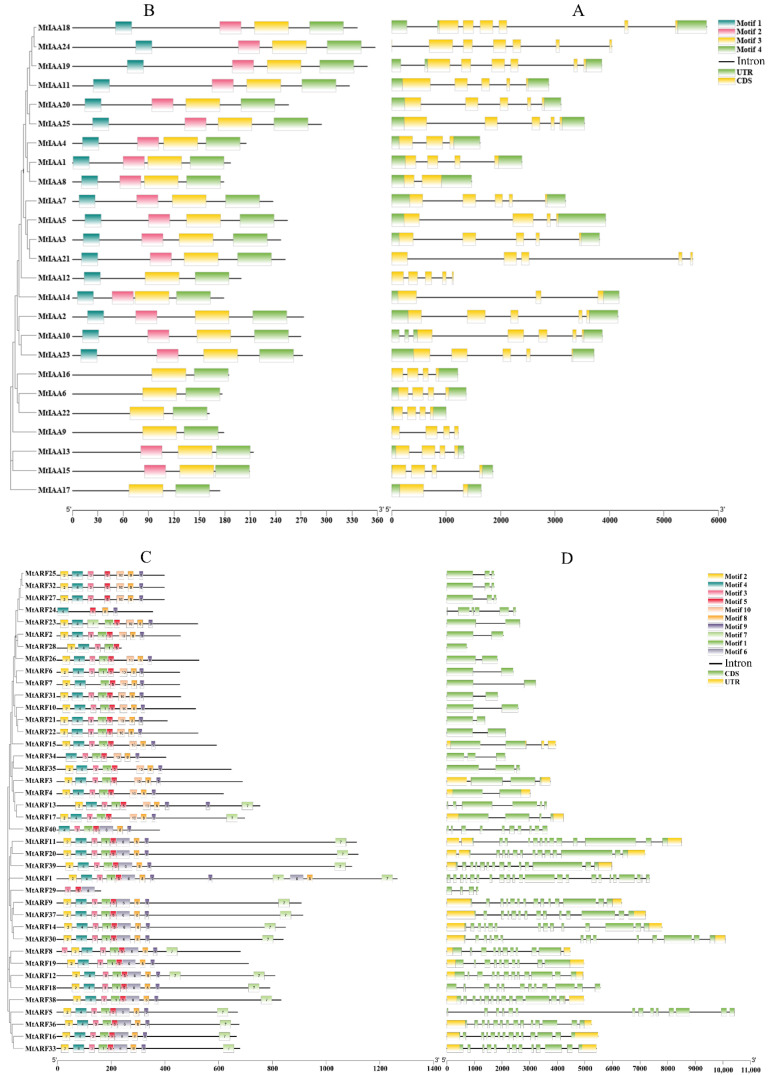
Characterization of MtIAA genes and MtARF proteins. (**A**,**D**) exon–intron structure distribution; (**B**,**C**) protein motif.

The arrangement of exons/introns was used for analysis of the gene structure of *MtIAAs* and *MtARFs*. One to six introns were found in the *Aux/IAA* members. Most of the *MtIAAs* had multiple introns. One intron was found in two *MtIAAs* (*MtIAA8*, *MtIAA17*); two introns in two *MtIAAs* (*MtIAA4*, *MtIAA14*); three introns in eight *MtIAAs;* four introns in ten *MtIAAs*; five in *MtIAA18* and *MtIAA19;* and six in *MtIAA24* (Figure 2A). Except for one member (*MtARF28*) without an intron, *MtARFs* had 1 to 21 introns. One intron was found in eleven *MtARFs*; two introns in six *MtARFs* (*MtARF3*, *MtARF25*, *MtARF27*, *MtARF32*, *MtARF34* and *MtARF35*); three in *MtARF17* and *MtARF29*; five in *MtARF13* and *MtARF24*; nine in *MtARF8*, *MtARF19* and *MtARF40*; eleven in *MtARF12* and *MtARF18*; twelve in *MtARF11*, *MtARF20*, *MtARF33* and *MtARF38*; thirteen in *MtARF5*, *MtARF9*, *MtARF14*, *MtARF16*, *MtARF30*, *MtARF36*, *MtARF37* and *MtARF39*; and twenty-one introns in *MtARF1* (Figure 2D).

A typical *Aux/IAA* gene contains four structure motifs (Ⅰ, Ⅱ, Ⅲ and Ⅳ) [3,36]. Most *MtIAA* members (17) contained four motifs. Two members (*MtIAA13* and *MtIAA15*) contained three motifs, missing motif I, while five *MtIAA* members (*MtIAA6*, *MtIAA9*, *MtIAA16*, *MtIAA17* and *MtIAA22*) contained only two motifs, missing motifs I and II (Figure 2B). A total of 10 conserved motifs in the *MtARFs* were identified. In fact, the B3 domain corresponded to motifs 2, 3 and 4; the *ARF* domain consisted of motifs 6, 8 and 9; and motifs 7 and 10 formed the CTD domain. In addition, B3 domain and the *ARF* domain constituted a conserved DBD structure. There were 15 *ARF* members (*MtARF1*, *5*, *9*, *11*, *12*, *14*, *16*, *18*, *20*, *30*, *33*, *36*, *37*, *38* and *39*), which contained all of the three domains. Except *MtARF29,* which only had the B3 domain, the rest of the 24 *MtARFs* lacked the CTD domain (Figure 2C).

### 2.4. Chromosomal Location and Synteny Analysis of MtIAA and MtARF Genes

The *MtIAA* and *MtARF* gene locations were mapped on chromosomes. All chromosomes had *MtIAA* and *MtARF* genes, except for chromosome 6, lacking *MtARF*. The largest number of *MtIAAs* (9) and *MtARFs* (12) were located on chromosomes 1 and 5, respectively, while the fewest of them were on chromosomes 6 and 3 (Supplementary Figure S1).

**Figure 3:**
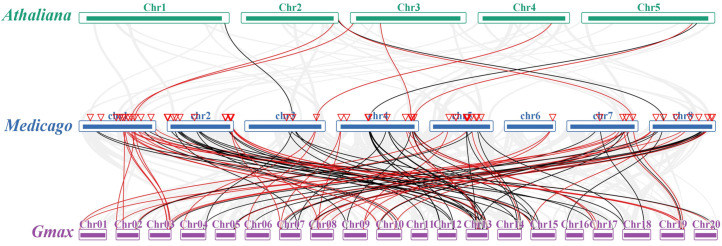
Synteny analysis of *IAA* and *ARF* genes in the genomes of *Medicago truncatula*, *Arabidopsis thaliana* and soybean. Green, blue and purple marks represent the *Athaliana*, *Medicago* and soybean chromosomes; the red and black marks represent *MtIAAs* and *MtARFs*, respectively.

Collinearity diagrams among *MtIAAs* and *MtARFs* were further analyzed. The results showed that some *MtIAA* and *MtARF* genes underwent gene duplication in the genomes of the *M. truncatula* genome; for example, *MtIAA3*/*MtIAA21*, *MtIAA6*/*MtIAA22*, *MtARF8*/*MtARF19*, *MtARF11*/*MtARF20* and *MtARF12*/*MtARF18* were pairs of segmental duplicates, respectively (Figure 3). Six pairs of homologous *IAAs* and three pairs of homologous *ARFs* were identified between *M. truncatula* and *A. thaliana*, respectively, but fifty-two pairs of orthologous *IAAs* and fifty-four pairs of orthologous *ARFs* were identified between *M. truncatula* and soybean, respectively (Figure 3 and Table S2). Six *MtIAAs* (*MtIAA5*, *MtIAA6*, *MtIAA13*, *MtIAA16*, *MtIAA17* and *MtIAA22*) and three *MtARFs* (*MtARF15*, *MtARF18* and *MtARF36*) had one homologous gene in *A. thaliana*, respectively, while four *MtIAAs* (*MtIAA2*, *MtIAA6*, *MtIAA11* and *MtIAA22*) and five *MtARFs* (*MtARF8*, *MtARF11*, *MtARF19*, *MtARF20* and *MtARF38*) had four homologous genes in soybean. In addition, four *MtIAAs* (*MtIAA3*, *MtIAA4*, *MtIAA21* and *MtIAA23*) and five *MtARFs* (*MtARF15*, *MtARF17*, *MtARF30*, *MtARF34* and *MtARF36*) had three homologous genes; the others had two homologous genes in soybean. The percentage of identity between pairs of paralogous MtIAA and MtARF proteins ranged from 65.54% to 71.04% and 65.52% to 78.91% in *M. truncatula*, respectively (Table S2). The identity of IAAs and ARFs between pairs of orthologous ranged 33.06% to 77.59% and 48.84% to 59.87% between *M. truncatula* and *A. thaliana*, respectively, while the identity of IAAs and ARFs between pairs of orthologous ranged from 43.93% to 80.75% and 38.40% to 90.91%, respectively, between *M. truncatula* and soybean. The high percentages of identity in IAAs and ARFs between *M. truncatula* and soybean suggest that MtIAA and MtARF protein sequences and functions were highly conserved and that *M. truncatula* is closely related to soybean.

### 2.5. Spatial and Temporal Expression of MtIAAs and MtARFs

**Figure 4:**
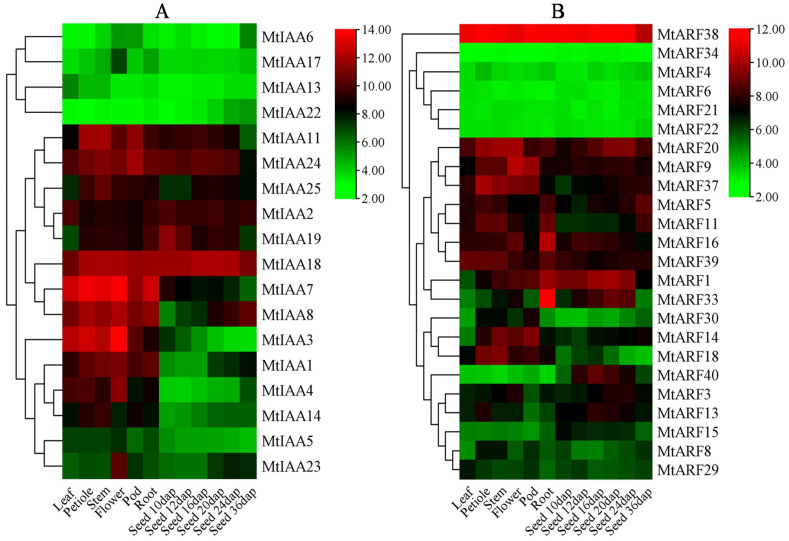
Spatial and temporal expression of *MtIAA* and *MtARF* genes. (**A**) *MtIAA*; (**B**) *MtARF*. *MtIAA* and *MtARF* expression levels are shown as the log2-based fluorescence intensity values from MtGEA (https://mtgea.Noble.orgv3/ (accessed on 24 January 2021)). DAP indicates days after pollination.

The spatial and temporal expression of MtIAAs and MtARFs were analyzed based on the microarray data (MtGEA, https://mtgea.Noble.orgv3/ (accessed on 24 January 2021)). The data of 18 *MtIAAs* and 24 *MtARFs* can be found in the dataset (Table S3). *MtIAA2*, *MtIAA7*, *MtIAA8*, *MtIAA11*, *MtIAA18* and *MtIAA24* were highly expressed in all tissues (roots, stems, leaves, flowers, petioles, pods and seeds) (Figure 4A and Table S3), indicating that they may have diverse functions. *MtIAA1*, *MtIAA3* and *MtIAA4* were mainly expressed in roots, stems, leaves, petioles and flowers. *MtIAA23* was preferentially expressed in flowers, while *MtIAA19* and *MtIAA25* were expressed in roots, petioles, stems, flowers, pods and seeds, but not in leaves.

*MtARF5*, *MtARF9*, *MtARF11*, *MtARF16*, *MtARF20*, *MtARF37*, *MtARF38* and *MtARF39* showed relatively high expression in all tissues (roots, stems, leaves, flowers, petioles, pods and seeds); among them, *MtARF38* had the highest expression (Figure 4B), indicating that *MtARF38* may play an important role in the regulation of growth and development in *M. truncatula*. *MtARF1* and *MtARF14* were mainly expressed in roots, stems, flowers, petioles, pods and seeds, but not in leaves. *MtARF18* was mostly expressed in roots, stems, leaves, flowers, petioles and pods, but not in seeds, while *MtARF40* was only expressed in seeds. *MtARF33* was majorly expressed in seeds and roots (Figure 4B and Table S3).

### 2.6. Analysis of cis-Acting Element in the Promoter Region of MtIAA and MtARF Genes

**Figure 5:**
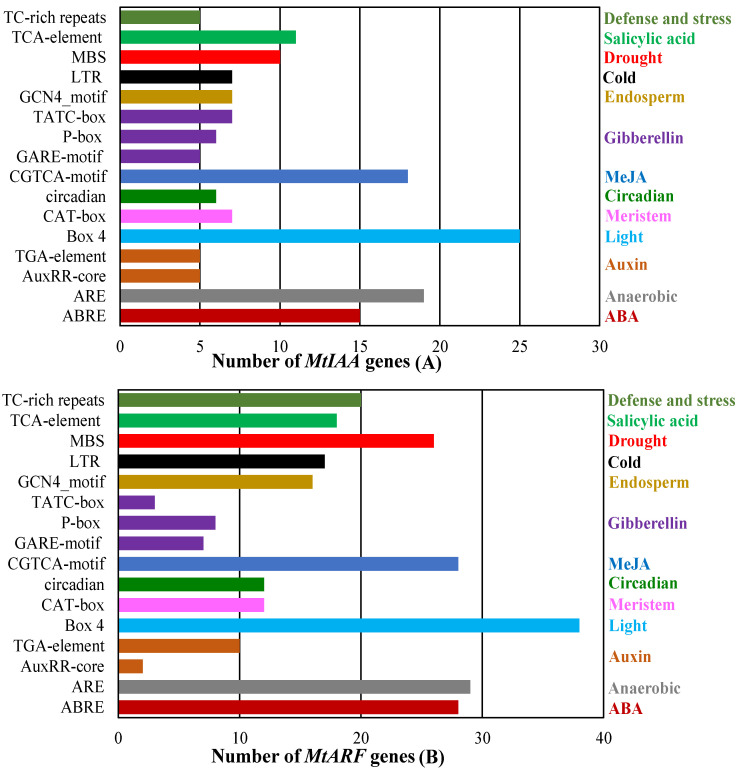
Number of *MtIAA* (**A**) and *MtARF* (**B**) genes containing various *cis*-acting elements. ABRE, ABA-responsive element; ARE, anaerobic induction element; AuxRR-core and TGA-element, *cis*-acting element involved in auxin-responsive element; CAT-box, *cis*-acting element involved in meristem expression element; circadian, *cis*-acting element involved in biologic rhythms; CGTCA-motif, *cis*-acting element involved in MeJA response; GARE-motif, P-box and TATC-box, *cis*-acting element involved in GA response; GCN4-motif, *cis*-acting element involved endosperm expression; LTR, *cis*-acting element involved in low-temperature response; MBS, *cis*-acting element involved in drought-inducibility; TCA-element, *cis*-acting element involved in SA response; TC-rich repeats, *cis*-acting element involved in defense and stress.

To understand the potential regulation of *MtIAA* and *MtARF* expression, a 2 kb promoter sequence of *MtIAAs* and *MtARFs* was analyzed; the results are listed in Table S4. Light (Box4), anaerobic (ARE), MeJA (CGTCA-moif), gibberellin (GARE-motif) and ABA (ABRE) response elements were abundant in the promoter of *MtIAAs* and *MtARFs*, while salicylic acid (TCA-motif), drought (MBS), auxin (AuxRR-core), cold (LTR), endosperm, meristem and circadian response elements were also found in the promoters (Figure 5A,B). Ten *MtIAAs* and twenty-six *MtARFs* had a drought response element; seven *MtIAAs* and seventeen *MtARFs* had a cold response element; and five *MtIAAs* and twenty *MtARFs* had a defense and stress response element. Seven *MtIAAs* and sixteen *MtARFs* had an endosperm response element, while six *MtIAAs* and twelve *MtARFs* had a circadian response element (Figure 5A,B). The results indicated that *MtIAAs* and *MtARFs* may be responsive to plant hormones, growth and development as well as various abiotic stresses.

### 2.7. Responses of MtIAAs and MtARFs to Salt, Drought and Cold

**Figure 6:**
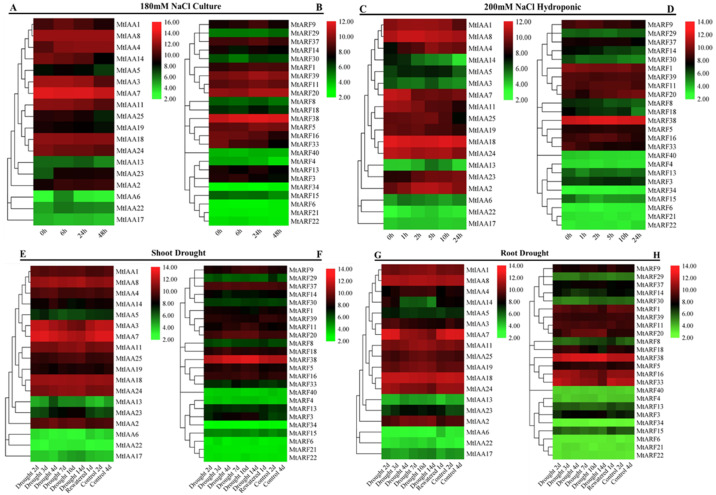
Responses of *MtIAAs* and *MtARFs’* expression to salt and drought stress. The microarray data were retrieved from *M. truncatula* Gene Expression Atlas (MtGEA, https://mtgea.Noble.orgv3/ (accessed on 24 January 2021)). (**A**,**B**) Two-day-old seedlings were treated by placing in 1/2 MS medium containing 180 mM NaCl for 0, 6, 24 and 48 h for salt stress. (**C**,**D**) Two-week-old seedlings were placed in a nutrient solution containing 200 mM NaCl for 1, 2, 5, 10 and 24 h as hydroponic treatment, with those growing in the nutrient solution as the control. (**E**–**H**) The 24-day-old seedlings growing in soil underwent withheld irrigation for 14 d of drought treatment before rewatering. *MtIAAs* and *MtARFs* expression levels are indicated as the log2-based fluorescence intensity values.

The responses of *MtIAAs* and *MtARFs’* expression to salt and drought stress were obtained from MtGEA (https://mtgea.Noble.orgv3/ (accessed on 24 January 2021)). *MtIAAs* and *MtARFs’* expression patterns were altered after salt and drought stress (Table S5). *MtIAA23* expression was upregulated after 6 h of salt treatment by placing on a 1/2 MS medium containing 180 mM NaCl. *MtIAA14* was downregulated with the extension of time, and *MtIAA5* was downregulated after 48 h of salt stress (Figure 6A). *MtARF14* was downregulated after 6 h, while *MtARF5*, *MtARF9*, *MtARF20*, *MtARF37* and *MtARF39* showed the highest expression after 14 h of salt stress (Figure 6B). In the hydroponic experiment, by treatment in a nutrient solution containing 200 mM NaCl, *MtIAA4* was upregulated within 5 h and downregulated at 10 h after treatment, whereas *MtIAA14* was downregulated continuously in response to the treatment (Figure 6C). Most of *MtARFs’* transcripts were invariable in the hydroponic treatment experiment, except for *MtARF33* and *MtARF14*, whose expression was decreased significantly (Figure 6D).

Compared to the induced expression of *MtIAA13*, *MtIAA23* and *MtARF3*, most of the *MtIAA* and *MtARF* transcripts were unaltered in shoots during drought treatment (Figure 6E,F). On the other hand, the *MtIAA14* and *MtARF14* transcripts in the roots were decreased after drought treatment followed by an increase after rewatering (Figure 6G,H), whereas *MtARF3* and *MtARF20* were induced by drought treatment followed by a decrease after rewatering (Figure 6H). The results indicated that some of *MtIAA* and *MtARF* genes may participate in salt and drought responses.

**Figure 7:**
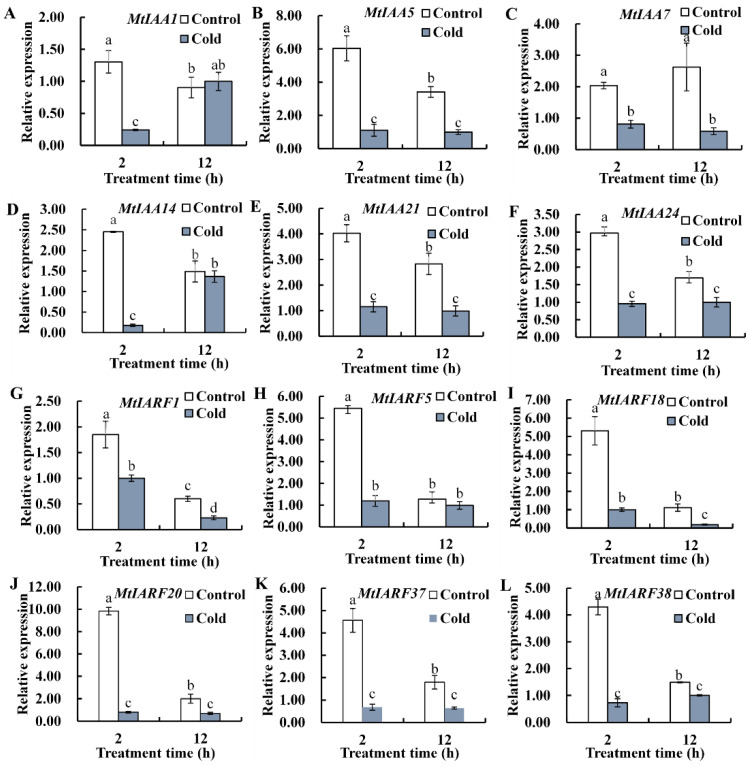
Relative expression of *MtIAA1* (**A**), *MtIAA5* (**B**), *MtIAA7* (**C**), *MtIAA14* (**D**), *MtIAA21* (**E**), *MtIAA24* (**F**), *MtARF1* (**G**), *MtARF5* (**H**), *MtARF18* (**I**), *MtARF20* (**J**), *MtARF37* (**K**) and *MtARF38* (**L**) in response to cold. The presented are means and standard errors of three independent experiments. The different letters in a column denote significant differences among the treatments at *p* < 0.05.

Six *MtIAAs* and six *MtARFs* that have LTR *cis*-acting element in the promoter regions were selected for analysis of gene expression in response to cold (Table S6). The *MtIAA1*, *MtIAA14* and *MtARF5* transcripts were significantly reduced after 2 h of cold treatment but showed no significant difference after 12 h. *MtIAA5*, *MtIAA7*, *MtIAA21*, *MtIAA24*, *MtARF1*, *MtARF18*, *MtARF20*, *MtARF37* and *MtARF38* transcript levels were significantly reduced at 2 and 12 h after cold treatment (Figure 7). The results suggest that *MtIAAs* and *MtARFs*, which have a LTR *cis*-acting element in the promoter regions, may participate in cold adaptation in *M. truncatula*.

### 2.8. Predicted MtIAA and MtARF Family Interaction Networks

**Figure 8:**
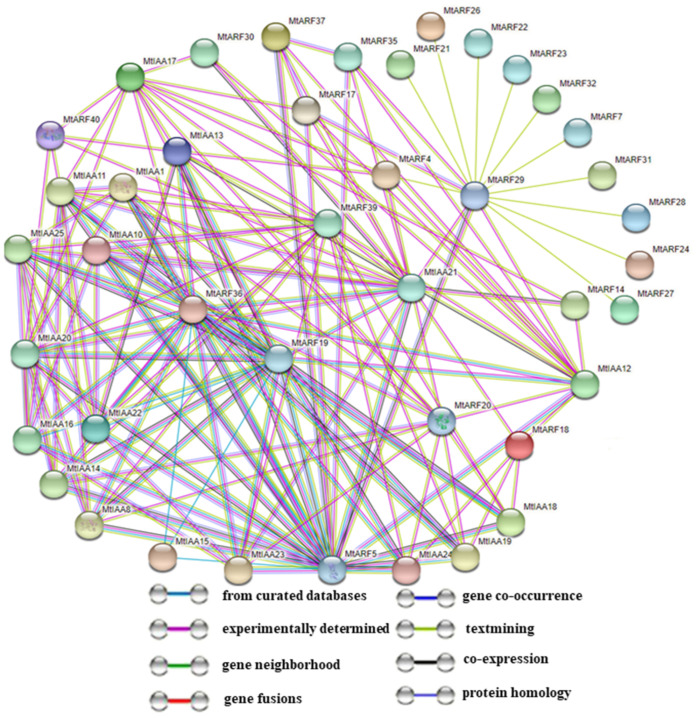
Predicted protein–protein interaction network of MtIAAs and MtARFs. The network contains 42 nodes (18 Aux/IAAs and 24 ARFs).

A protein–protein interaction network between MtIAAs and MtARFs was predicted by the STRING (https://www.string-db.org/ (accessed on 20 March 2021)) software. 18 MtIAAs and 24 MtARFs were found to form a protein–protein interaction network (Figure 8). The results showed that some MtARF proteins could interact with multiple MtIAAs, while some MtIAAs could interact with multiple MtARF. It is notable that four MtARFs (MtARF5, MtARF19, MtARF36 and MtARF39) that function as activators may interact strongly with most of MtIAA proteins. In addition, MtARF29 may interact with MtIAA12 and MtIAA21 as well as multiple MtARFs. Moreover, a lot of *MtARF* genes showed co-expression correlation, indicating that these genes might be involved in the same regulatory pathway. For example, *MtARF5* had high co-expression levels with 11 *MtIAAs* and 3 *MtARFs*, and *MtARF19* with 10 *MtIAAs* and 2 *MtARFs*, indicating that *MtARF5* or *MtARF19* might be a key regulator among the 40 *MtARFs* (Figure 8 and Figure S2).

## 3. Discussion

Auxin plays a critical role in controlling plant growth and developmental and physiological processes, while IAA and ARF are key components in auxin signaling for regulating downstream reactions [4]. The numbers of *IAA* and *ARF* members are different among plants species; for example, there are 29 *IAAs* and 23 *ARFs* in *Arabidopsis* [6,20], 31 *IAAs* and 25 *ARFs* in rice [19,22], and 63 *IAAs* and 55 *ARFs* in soybean [27,34]. A total of 17 *IAA* and 24 *ARF* genes were reported in *M. truncatula* [32,33], while a total of 25 *MtIAA* and 40 *MtARF* genes were identified in this study based on the updated genome data. *MtIAAs* and *MtARFs* showed extensive variations in ORF length, predicted MW and pI, which was also observed in rice *IAAs* [19] and *Brachypodium distachyon ARFs* [30]. The variations implied that the diverse MtIAA and MtARF proteins might function under different microenvironments. All MtIAAs and MtARFs had negative GRAVY, suggesting that they are hydrophilic proteins. Like those in other plant species [28,29,37], most *MtIAA* and *MtARF* genes have multiple introns.

A typical Aux/IAA protein contains four conserved domains designated as I, II, III and IV [3,35]. A total of 17 MtAux/IAA proteins contained 4 domains, while the others lost at least 1 domain (Figure 2B). MtIAA13 and MtIAA15 proteins lost domain I, indicating that they might experience a loss in capacity in recruiting TPL co-repressors and thus, lost the function as a repressor in auxin signaling. In addition, MtIAA12 protein, like *AtIAA20* [4], lost domain II, indicating that it should not be degraded under increased levels of auxin [4]. Recent studies have shown that, instead of degrading non-canonical Aux/IAA proteins by TIR1/AFB, auxin stabilizes non-canonical Aux/IAA proteins by phosphorylation of upstream protein kinases; for instance, auxin regulates the stability of non-canonical AtIAA32 and AtIAA34 proteins through transmembrane kinases (TMK), and then regulates gene expression through ARF transcription factors to mediate the differential growth during apical-hook development [38]; meanwhile, auxin also regulates the stability of non-canonical AtIAA33 protein through MITOGEN-ACTIVATED PROTEIN KINASE 14 (MPK14) and does not affect *AtIAA33* gene expression [15]. Another explanation is that the Aux/IAA proteins are too low in the tissues to be not able to affect plant growth and development [35], even some of the deduced sequences might be pseudogenes, because no information about expression is available for several of them [36]. The expression level of *MtIAA6*, *MtIAA17* and *MtIAA22* genes lacking domain II was very low in all major tissues, compared with the canonical *Aux/IAA* genes, while the others (*MtIAA9*, *12* and *16*) showed no available information (Figure 4A), which was consistent with those in *Brassica napus* lacking domain II [35]. The others MtIAAs (MtIAA6, MtIAA9, MtIAA16, MtIAA17 and MtIAA22) lost both domains I and II, indicating that they could neither be a repressor nor be rapidly degraded in auxin signaling. The truncated Aux/IAA proteins also exist in multiple plant species. Domains I and II are lost in AtIAA29 and AtIAA33 in *Arabidopsis*, and OsIAA4, OsIAA27, OsIAA28 and OsIAA29 in rice [6,19]; Domain II is lost in CaIAA5, -11, -12, -16, -17 and -19 in chickpea; and GmIAA5, -6, -13, -23, -31, -35, -37, -39, -40, -42, -53 and -60 in soybean [34]. Domain III or IV is lost in PeIAA1, -18, and -24 proteins in moso bamboo [39]. Thus, the variations in these domains are associated with diverse functions of Aux/IAA in the auxin signaling pathway.

A typical ARF protein contains three domains, designated as DBD, MR and CTD [3]. ARF proteins rely on the DBD to bind specifically to auxin response elements (AuxRE: TGTCTC) in the promoters of auxin responsive genes [9]. The amino acid composition of the MR region depends on it as an activator or repressor [33]. The CTD is involved in homo- and hetero-interactions among ARFs [23]. A total of 14 MtARF proteins have complete domains, while MtARF29 lack MR and CTD domains and the others lack the CTD domain (Figure 2C). Eight MtARFs (MtARF1, -9, -11, -14, -20, -30, -37 and -39) are predicted to be transcriptional activators based on the fact that glutamine (Q), serine (S) and leucine (L) are enriched in the MR domain, while seven (MtARF5, -12, -16, -18, -33, -36 and -38) are putative transcriptional repressors because S, L, proline (P) and glycine (G) are enriched in the MR region. A total of 24 MtARF proteins, rich in S, L, proline (P) and glycine (G) in the MR region, are putative transcriptional repressors that lack a CTD. This means that the ratio of activator/repressor numbers of MtARFs is 0.26, which is consistent with previous reports [33]. Our investigation provides insight into understanding the potential role of *MtARF* genes in the regulation of plant developmental processes and responses to environmental stresses. Canonical auxin responsive transcription factor ARF family proteins bind to the promoter region of *Aux/IAA* genes through their CTD domain and are regulated by TIR1/AFB receptors [3]. The loss of CTD in MtARFs revealed that they may function in an auxin-independent manner. AtARF3 that lacks the CTD domain did not bind with the elements of the canonical TIR1/AFB signaling pathway and functions, independent of the TIR1/AFB receptor [40].

TBtools (v1.09854, Chengjie Chen, Guangzhou, China) software was used for analyzing the synteny of IAA or ARF genes among *M.truncatula*, *A.thaliana* and soybean. A total of 6 MtIAA-AtIAA pairs but 52 MtIAA-GmIAA pairs were observed among Aux/IAA family, and 3 MtARF-AtARF pairs but 54 MtARF-GmARF pairs among the ARF family. The results support that *M. truncatula* is phylogenetically closer with soybean than with *Arabidopsis*. Two pairs of MtIAAs (*MtIAA3*/*MtIAA21*, *MtIAA6/MtIAA22*) and three pairs of MtARFs (*MtARF8/MtARF19*, *MtARF11/MtARF20* and *MtARF12/MtARF18*) belong to segmental duplication, indicating that *M. truncatula* has undergone local gene duplication and shares an ancient round of gene duplication with other legume species [31].

The spatial expression of genes is related to their potential functions. Some *MtIAA* and *MtARF* genes showed specific and overlapping expression patterns in various tissues and developmental stages, implying that they may have specific functions. Seven *MtIAAs* (*MtIAA3*, *MtIAA4*, *MtIAA7*, *MtIAA8*, *MtIAA17*, *MtIAA18* and *MtIAA23*) were highly expressed in floral organs (Figure 4A), and four *MtARFs* (*MtARF1*, *MtARF16*, *MtARF33* and *MtARF38*) were highly expressed in roots, suggesting that they are associated with flowering and root regulation, respectively. *AtIAA7*, the homolog of *MtIAA7*, is involved in the regulation of flowering time via the negatively regulating expression of *GA20ox1* and *GA20ox2* under short-day light conditions in *Arabidopsis* [41]. *AtARF7*, the homolog of *MtARF20*, is involved in the regulation of lateral root formation via activating *LBD*/*ASL* genes in *Arabidopsis* [42]. Some Aux/IAA and ARF proteins regulate gene transcription during leaf growth in the tomato, such as *SlIAA1*, *SlIAA7*, *SlIAA19* and *SlIAA24* [16], while 12 IAA genes were upregulated in leaves in *Brassica napus* [35]. Five *PeIAAs* (*PeIAA1*, *PeIAA2*, *PeIAA6*, *PeIAA8* and *PeIAA16*) and four *PeARFs* (*PeARF8*, *PeARF14*, *PeARF18* and *PeARF19*) were highly expressed in shoots [38], and *GmIAA45* and *GmIAA51* transcripts were found in soybean shoots [34]. 

Some promoter elements (LTR, MBS, ABRE, CGTCA-motif, AuxRR-core, Box4, TCA-element and TATC-box) were enriched multiple times in the promoter regions of *MtIAA* and *MtARF* genes. Some *IAA* and *ARF* genes participated in hormonal or abiotic stresses responses when specific cis-elements were found in their promoter regions, such as *AtIAA7* [41], *SlIAA2*, *SlIAA11*, *SlIAA17*, *SlIAA19* and *SlIAA29* [16], *OsIAA20* [43], *OsARF16* [24], *SlARF2B,* SlARF5 and *SlARF9A* [44], BdARF17 and BdARF23 [30]. Two *MtARF* genes (*MtARF14* and *MtARF18*) and more than half of the *MtIAA* genes showed extensive responses to salt stresses. Four *MtIAA* genes (*MtIAA2*, *MtIAA7*, *MtIAA14* and *MtIAA23*) and *MtARF* genes (*MtARF3*, *MtARF11*, *MtARF14* and *MtARF20*) showed extensive responses to drought stresses, respectively. Similarly, many *IAA* and *ARF* genes in other plant species were induced by drought treatment [29,30,45,46]. Transcripts of *BdARF5*, *BdARF12*, *SbIAA1*, *SbIAA26* and *SbARF3* were up-regulated substantially following salt stress [30,46]. Six *MtIAA* genes showed downregulation in response to cold treatment. The downregulation of *Aux/IAAs* leads to the release of the inhibited ARF gene so that ARF regulates the expression of downstream auxin response genes and causes a series of auxin-related responses [3,23]. In addition, six *MtARF* genes were also downregulated after cold treatment. Twenty *BdARFs* and *CaIAA3* and *CaIAA7* transcripts were induced after cold treatment in *Brachypodium distachyon* and chickpea, respectively [30,34]. The responses of *MtIAAs* and *MtARFs* to drought, salt and cold suggest that *MtIAAs* and *MtARFs* are involved in abiotic stress adaptation in *M. truncatula*.

Protein-protein interactions are critically important to many processes, such as signal transduction and regulation of gene expression. Auxin responses are mediated by interaction between ARF and Aux/IAA proteins [7]. Therefore, it is significant to study the interaction between IAA and ARF in *M. truncatula*. In this study, the protein-protein interaction networks included 161 interaction combinations between 18 *MtIAAs* and 24 *MtARFs* (Figure 8). A total of 213 specific interactions between 19 ARFs and 29 Aux/IAA were identified in *Arabidopsis*, and up to 70% of ARF interacted with Aux/IAA factors by integrating co-expression maps with protein-protein interaction data [47]. Moreover, we observed that a single ARF protein could interact with multiple Aux/IAA and the reverse is also true; for example, MtARF29 interacted with MtIAA12 and MtIAA21 as well as a large number of MtARFs, which is consistent with the observation that SlARF2A interacts with five SlIAAs, and SlARF6A interacts with at least 11 SlIAAs in the tomato plant [48]. Similarly, transcriptional activators AtARF5, AtARF6, AtARF7, AtARF8 and AtARF19 interacted with almost all Aux/IAA proteins in *Arabidopsis* [47]. The predicted interaction networks and the co-expression network in this study provide clues for further investigations on the regulation of MtAux/IAA-MtARF on the growth, development and adaptation to environmental stresses in *M. truncatula*.

## 4. Material and Methods

### 4.1. Identification of IAA and ARF Genes in M. truncatula

The protein sequences of *M. truncatula* Mt4.0v1 were downloaded from Phytozome 12 (https://phytozome.jgi.doe.gov/pz/portal.html (accessed on 22 October 2020)). The reported *IAA* and *ARF* gene sequences in *A. thaliana* were downloaded from the TAIR database (https://www.arabidopsis.org/ (accessed on 22 October 2020)) and as queries for BLASTP search. The potential *IAA* and *ARF* genes in *M. truncatula* were searched via the NCBI database. Moreover, the protein sequences of GmIAAs and GmARFs were downloaded from SoyBase (https://www.soybase.org/ (accessed on 22 October 2020)) and used to search against the *M. truncatula* proteome. According to the main characteristics of the Aux/IAA protein family (Pfam:02309 AUX/IAA family) and *ARF* gene family (Pfam:02309: AUX/IAA family; Pfam 06507: Auxin_resp; Pfam 02362: B3 DNA binding domain) [32,33], *IAA* and *ARF* candidate genes were screened to distinguish the IAA and ARF homologous genes in *M. truncatula*. All generated non-redundant protein sequences were detected by SMART (http://smart.embl-heidelberg.de/ (accessed on 30 October 2020)) and InterProScan (http://www.ebi.ac.uk/interpro/ (accessed on 30 October 2020)) for the presence of major characteristic structures.

### 4.2. Analysis of Conserved Domain, Gene Structure and Characterization of MtIAA and MtARF Genes

Using the ProtParam tool of ExPASy (http://web.expasy.org/protparam/ (accessed on 28 October 2020)), we analyzed the physical and chemical characteristics, containing the molecular weight (MW), theoretical point (PI) and grand average of hydropathicity (GRAVY) of MtIAA and MtARF proteins. Using the TBtools (Toolbox for Biologists) program with default parameters, we analyzed the exon–intron structure of *MtIAA* and *MtARF* genes [49]. Using the MEME tool (http://meme-suite.org/tools/meme (accessed on 4 November 2020)), we analyzed the conserved motifs, with the minimum width of motifs as 10, the maximum width of motifs as 40 and the other parameters as default values.

### 4.3. Phylogenetic Relationships of MtIAA and MtARF Proteins in M. truncatula, Arabidopsis and Soybean

Using ClustalX with default parameters, we performed the multiple alignments for MtIAA and MtARF proteins, respectively. Using MEGA X with the maximum-likelihood (ML) method and 1000 bootstrap replicates, we analyzed the phylogenetics of IAAs and ARFs in *M. truncatula*, *Arabidopsis* and soybean [50].

### 4.4. Chromosomal Locations of MtIAA and MtARF Genes

Using the sequence of *MtIAA* and *MtARF* genes, we searched their chromosomal locations in *M. truncatula* genome databases, such as Phytozome 12 (https://phytozome.jgi.doe.gov/pz/portal.html (accessed on 22 October 2020)). Using the TBtools software, we analyzed the chromosomal locations and homologous relationship of *MtIAA* and *MtARF* genes [49]. Using the Multiple Collinearity Scan toolkit (MCScanX), we analyzed the gene duplication events [49].

### 4.5. Analysis of cis-Acting Elements of MtIAA and MtARF Genes

Genomic DNA sequences of 2000 bp upstream of each *MtIAA* and *MtARF* transcription start site were obtained from the Phytozome database (https://phytozome.jgi.doe.gov/pz/portal.html (accessed on 22 October 2020)); using the PlantCARE database (http://bioinformatics.psb.ugent.be/webtools/plantcare/html/ (accessed on 28 January 2021)), we analyzed the *cis*-acting elements [51].

### 4.6. Analysis of Microarray Expression Profile

The genome-wide microarray data of *M. truncatulain* different tissues at various developmental stages and the response to drought and salt were searched from *M. truncatula* Gene Expression Atlas (MtGEA, https://mtgea.noble.org/v3/ (accessed on 24 January 2021)). Using the TBtools (v1.09854, Chengjie Chen, Guangzhou, China) software, we analyzed the transcript data and the normalized expression data of *MtIAAs* and *MtARFs* to generate heat [49].

### 4.7. Analysis of Relative Expression of MtIAAs and MtARFs in Response to Cold

The seeds of *M. truncatula* (R108) were treated with sandpaper to break the physical dormancy, and then placed on wet paper towel to absorb the water. After placing in a freezer at four °C for three days for vernalization treatment, the seeds were moved to room temperature for germination, followed by sowing in a plastic pot filled with soil. The plants were grown in a greenhouse for four weeks under natural light with temperature ranging from 20 to 28 °C, and then transferred to a growth chamber at 5 °C with a 12 h photoperiod under 200 μmol m^−2^ s^−2^ light for cold treatment, while those in a growth chamber at room temperature were used as the control. Leaves (0.1 g) were harvested for isolation of total RNA, using RNAprep pure Plant Kit (TIANGEN, Beijing, China), according to the manufacturer’s instructions. The cDNA synthesis and quantitative RT-PCR (qRT-PCR) were conducted as previously described [52]. Relative expression was calculated by 2^−∆∆Ct^. The *MtActin* gene was used as the internal control. The primers were listed in Table S8.

### 4.8. Predicted Protein Interaction Network and Co-Expression Network Construction

The interacting networks of MtIAA and MtARF proteins were integrated in the STRING (https://www.string-db.org/ (accessed on 20 March 2021)) software, and the co-expression network data were exported from STRING and calculated by Microsoft Excel 2019.

## 5. Conclusions

A total of 25 *MtIAA* and 40 *MtARF* genes were identified in *M. truncatula* based on version Mt4.0v1 of the *M. truncatula* genome database. Analysis of the intron-exon structure revealed that *IAA* and *ARF* gene families are evolutionarily conserved. Synteny analysis showed that tandem duplication probably participated in driving the *MtIAA* and *MtARF* genes’ evolution. *MtIAA* and *MtARF* genes were expressed in all organs detected, while some genes showed tissue-specific expression. The *cis*-acting elements responsive to plant hormones were enriched in the promoter of *MtIAAs* and *MtARFs*, while salicylic acid (TCA-motif), drought (MBS), cold (LTR), endosperm, meristem and circadian response elements were found in the promoter of some *MtIAAs* and *MtARFs*. Many *MtIAAs* and *MtARFs* were regulated by drought, salt and cold. The protein-protein interaction predicted 161 interaction combinations between 18 *MtIAAs* and 24 *MtARFs*. The study provides a valuable resource for further studies on the biological functions of MtIAA and MtARF genes in the regulatory mechanisms of auxin-related pathways in *M. truncatula*.

## Figures and Tables

**Table 1:** Information on MtIAA proteins.

Name	Locus ID	ORF (bp)	A.A.	MW (KDa)	pI	GRAVY
MtIAA1	Medtr1g040675	561	186	20.76	6.75	−0.554
MtIAA2	Medtr1g069495	819	272	30.11	7.15	−0.814
MtIAA3	Medtr1g070520	738	245	27.48	7.57	−0.689
MtIAA4	Medtr1g070830	615	204	22.91	6.20	−0.732
MtIAA5	Medtr1g080860	762	253	27.51	6.75	−0.512
MtIAA6	Medtr1g085750	531	176	19.80	5.56	−0.535
MtIAA7	Medtr1g093240	711	236	25.72	8.60	−0.552
MtIAA8	Medtr1g093350	537	178	19.98	7.68	−0.752
MtIAA9	Medtr1g109510	537	178	20.19	4.77	−0.493
MtIAA10	Medtr2g100780	810	269	29.96	8.86	−0.863
MtIAA11	Medtr2g101500	981	326	35.36	8.11	−0.539
MtIAA12	Medtr2g102490	597	198	22.50	8.80	−0.529
MtIAA13	Medtr3g106850	642	213	24.73	9.00	−0.883
MtIAA14	Medtr4g011880	537	178	20.14	6.21	−0.598
MtIAA15	Medtr4g115075	627	208	24.00	6.16	−0.853
MtIAA16	Medtr4g124300	555	184	20.88	6.51	−0.497
MtIAA17	Medtr4g128070	522	173	19.22	7.78	−0.524
MtIAA18	Medtr5g030710	1008	335	36.26	8.52	−0.468
MtIAA19	Medtr5g067350	1044	347	38.01	8.62	−0.567
MtIAA20	Medtr6g488150	765	254	28.32	8.43	−0.580
MtIAA21	Medtr7g096090	753	250	27.90	5.36	−0.558
MtIAA22	Medtr7g110790	486	161	18.19	5.68	−0.358
MtIAA23	Medtr8g014520	816	271	30.12	8.28	−0.755
MtIAA24	Medtr8g067530	1071	356	38.67	6.38	−0.455
MtIAA25	Medtr8g103030	882	293	31.89	8.04	−0.509

**Table 2:** Information on MtARF proteins.

Name	Locus ID	ORF (bp)	A.A.	MW (KDa)	pI	GRAVY
MtARF1	Medtr1g024025	3798	1265	141.16	5.26	−0.439
MtARF2	Medtr1g058210	1380	459	51.30	8.32	−0.383
MtARF3	Medtr1g064430	2070	689	76.95	6.69	−0.475
MtARF4	Medtr1g094960	1860	619	68.96	7.53	−0.477
MtARF5	Medtr2g005240	2016	671	74.98	5.71	−0.570
MtARF6	Medtr2g006270	1371	456	51.92	8.67	−0.426
MtARF7	Medtr2g006380	1371	456	52.06	6.92	−0.464
MtARF8	Medtr2g014770	2049	682	74.20	6.11	−0.323
MtARF9	Medtr2g018690	2727	908	100.83	6.14	−0.480
MtARF10	Medtr2g024430	1548	515	57.55	6.20	−0.372
MtARF11	Medtr2g043250	3345	1114	124.37	5.99	−0.721
MtARF12	Medtr2g093740	2433	810	90.08	7.24	−0.430
MtARF13	Medtr2g094570	2268	755	84.01	8.64	−0.379
MtARF14	Medtr3g064050	2550	849	94.27	5.92	−0.503
MtARF15	Medtr3g073420	1782	593	64.87	6.15	−0.218
MtARF16	Medtr4g021580	2004	667	74.19	6.22	−0.470
MtARF17	Medtr4g058930	2097	698	77.25	7.21	−0.340
MtARF18	Medtr4g060460	2379	792	88.10	6.36	−0.455
MtARF19	Medtr4g088210	2139	712	79.09	6.50	−0.469
MtARF20	Medtr4g124900	3363	1120	125.41	6.08	−0.687
MtARF21	Medtr5g040740	1233	410	45.10	8.42	−0.414
MtARF22	Medtr5g040880	1575	524	58.20	6.33	−0.385
MtARF23	Medtr5g060630	1572	523	58.09	5.14	−0.407
MtARF24	Medtr5g060770	1071	356	39.51	5.20	−0.264
MtARF25	Medtr5g060780	1200	399	44.40	6.02	−0.321
MtARF26	Medtr5g061220	1587	528	58.55	6.37	−0.363
MtARF27	Medtr5g061890	1200	399	44.14	5.86	−0.390
MtARF28	Medtr5g062970	720	239	26.85	7.23	−0.042
MtARF29	Medtr5g074840	492	163	18.06	8.87	0.258
MtARF30	Medtr5g076270	2526	841	93.28	5.93	−0.472
MtARF31	Medtr5g082140	1383	460	51.65	6.47	−0.431
MtARF32	Medtr5g460920	1200	399	44.45	5.86	−0.306
MtARF33	Medtr7g062540	2043	680	76.17	5.74	−0.545
MtARF34	Medtr7g101275	1218	405	46.08	8.10	−0.491
MtARF35	Medtr7g101280	1947	648	72.45	8.41	−0.539
MtARF36	Medtr8g027440	2034	677	75.74	6.18	−0.502
MtARF37	Medtr8g079492	2745	914	102.26	6.08	−0.605
MtARF38	Medtr8g100050	2502	833	93.08	6.06	−0.704
MtARF39	Medtr8g101360	3291	1096	121.07	6.23	−0.522
MtARF40	Medtr8g446900	1146	381	43.48	5.86	−0.078

A.A. indicates the number of amino acids; pI indicates theoretical isoelectric point; MW indicates theoretical molecular weight; GRAVY indicates grand average of hydropathicity.

## Data Availability

Not applicable.

## Supplementary Materials

The following are available online at https://www.mdpi.com/article/10.3390/ijms221910494/s1.
