# Supplementary figures and images for: Genome-Wide Identification and Expression Analysis of the Aux/IAA and Auxin Response Factor Gene Family in Medicago truncatula
Source: Int J Mol Sci. 2021 Sep 28;22(19):10494. doi: 10.3390/ijms221910494 (PMC8532000; doi:10.3390/ijms221910494)

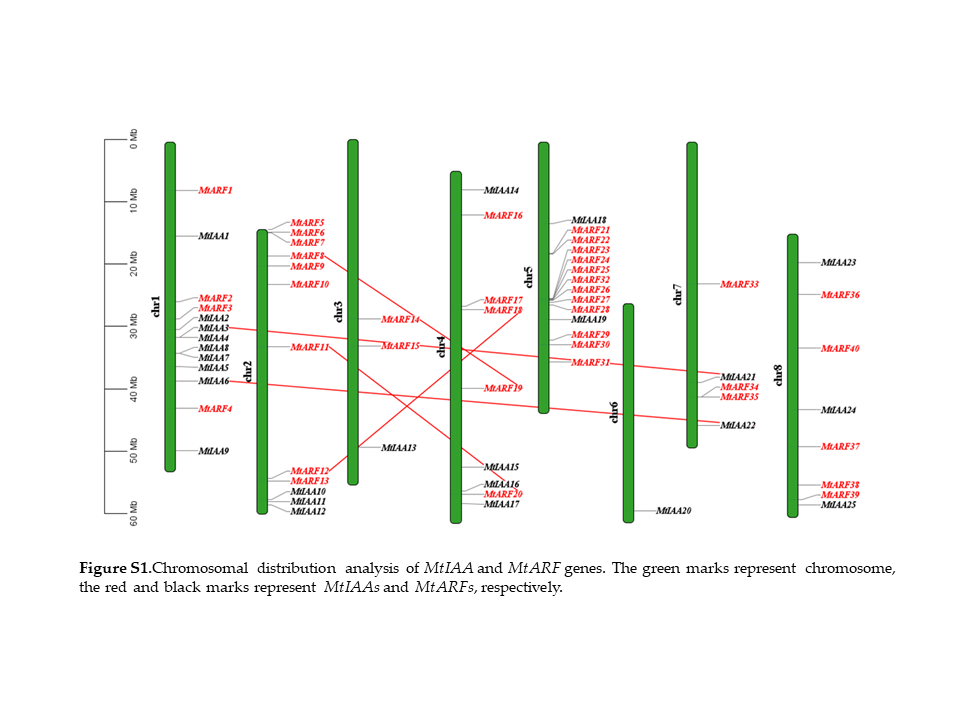

Supplement: Supplementary file 1 [file ijms-22-10494-s001.zip › Supplementary/Figure S1 Chromosomal distribution analysis of MtIAA and MtARF genes.tif]

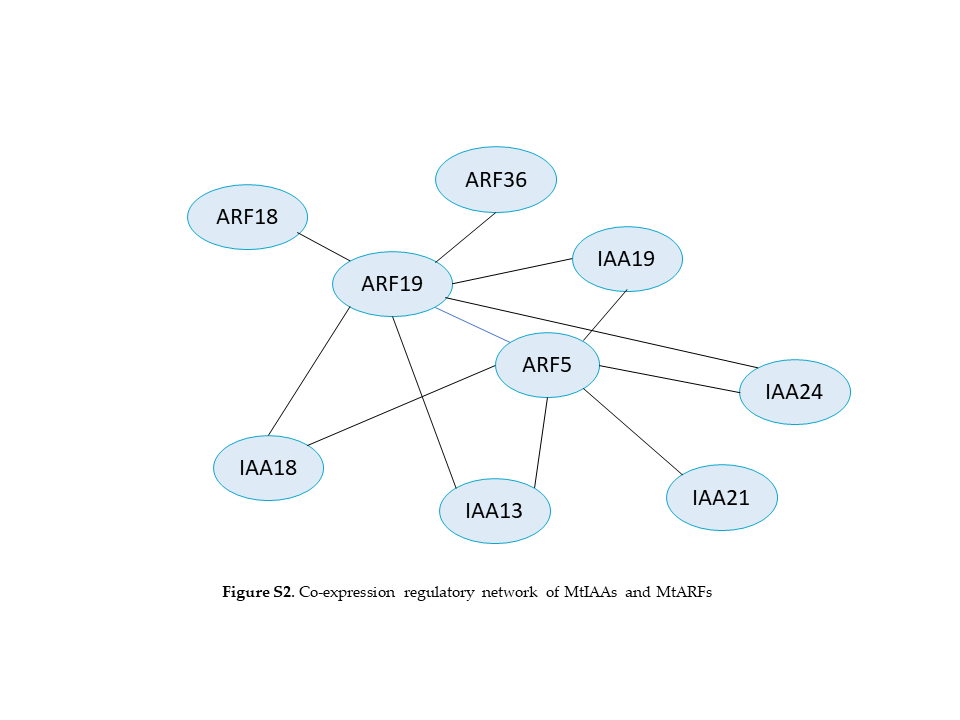

Supplement: Supplementary file 1 [file ijms-22-10494-s001.zip › Supplementary/Figure S2 Co-expression regulatory network of MtIAAs and MtARFs.tif]
